# UV-Induced Self-Renewing Wear-Resistant Flexible Polymer from a Polyurethane/Thiol–Ene Hybrid System

**DOI:** 10.3390/ma19071366

**Published:** 2026-03-30

**Authors:** Wenhao Wang, Yanhui Niu, Jiuguang Geng, Yu Zeng, Peng Yang, Zewen He, Xu Li, Bin Luan

**Affiliations:** 1 School of Materials Science and Engineering, Chang’an University, Xi’an 710061, China; 2022031013@chd.edu.cn (W.W.); zengyu@cndong.com (Y.Z.); yy92647364@163.com (P.Y.); hezewen@chd.edu.cn (Z.H.); 2 Great Bay Institute for Advanced Study, Great Bay University, Dongguan 200438, China; 3 School of Transportation Engineering, Chang’an University, Xi’an 710061, China; light@chd.edu.cn; 4 Yuzhongqing Waterproof Technology Group Co., Ltd., Xi’an 710200, China; 13021271550@163.com

**Keywords:** thiol–ene chemistry, polyurethane hybrid, dual-network, self-renewing surface, wear-resistant

## Abstract

**Highlights:**

- A sequential thermal–UV curing strategy is developed to achieve long-term wear resistance in flexible polymer materials.
- The self-renewing surface layer demonstrates excellent durability.
- The thiol–ene component strengthens the surface properties and bulk elasticity.
- The dual network underpins these properties and enables dual-shape memory.

**Abstract:**

Developing materials that simultaneously exhibit bulk elasticity and a durable, self-renewing surface is a persistent challenge, as traditional fillers often impair flexibility and sacrificial coatings fail under repeated strain. This paper presents an innovative thiol–ene/polyurethane hybrid system, fabricated via a sequential thermal–UV curing process, which decouples the properties of the highly elastic bulk from those of the robust surface layer. The resulting bulk elastomer achieves an outstanding combination of high strength (20.9 MPa) and exceptional extensibility (990% elongation at break). Crucially, the UV-crosslinked surface forms a dense, abrasion-resistant shield that reduces friction-induced mass loss by 81% compared to the bulk material. This surface layer also exhibits a unique self-renewing capability, effectively restoring its protective function over at least three abrasion cycles and reducing mass loss by 57% after the first recovery cycle relative to an unprotected control. Dynamic mechanical analysis validates the distinct dual-network structure, evidenced by two well-separated glass transition temperatures, which underpin the material’s pronounced shape memory effect. This work provides a design paradigm for creating flexible and durable polymer systems with independently tailored bulk and surface properties, offering significant potential for applications in artificial skin and demanding flexible components.

## 1. Introduction

Polyurethane elastomers are a class of polymers recognized for their outstanding mechanical properties and high deformability, holding broad application prospects across various fields [1,2]. In practical applications, these materials are often required to combine high flexibility with excellent surface wear resistance to withstand mechanical and tribological challenges in complex environments [3,4,5]. While high deformability can be achieved through molecular design, wear resistance has traditionally relied on the addition of fillers or the application of external coatings [6,7]. However, such strategies often compromise material flexibility, and externally applied wear-resistant layers are prone to cracking or delamination under large deformations due to modulus mismatch and interfacial stress concentration [8,9]. This conflict arises because, in homogeneous systems, wear resistance is generally positively correlated with a high modulus. As a result, high-modulus surfaces tend to experience greater stress under deformation than softer polymers, increasing the risk of instability at high modulus interfaces and adhesion interfaces with other materials under the same amount of deformation. Therefore, developing a strategy to decouple surface wear resistance from bulk modulus within homogeneous polymers—integrating a soft, deformable bulk with durable, wear-resistant surfaces—is a valuable approach to balancing the performance of special structural polymers.

An ideal solution would enable simultaneous tuning of surface wear resistance and bulk softness within a single material system, thereby decoupling surface durability from high bulk modulus. A hybrid system based on thiol–ene and amino–ester chemistry offers a promising pathway: the system first undergoes thermal curing to form a highly elastic polyurethane network, followed by a UV-induced thiol–ene click reaction in the surface region to construct a wear-resistant layer in situ. This surface layer not only significantly enhances resistance to abrasion and environmental erosion [10] but also exhibits a photo-triggered self-renewal capability—surface wear exposes unreacted photoactive groups, enabling continued recovery of surface functionality through re-curing. Meanwhile, the remaining unreacted photoactive components in the bulk act as molecular lubricants, weakening hydrogen bonding and microphase separation in the polyurethane, thereby softening the polymer matrix and increasing its deformability and ultimate elongation at break.

Conventional thermal–photo hybrid systems (including freeradical, cationic, and hybrid photopolymerization systems) typically employ a photo-first-thermal-later sequence [11,12,13,14], making them unsuitable for applications requiring the prior formation of a stable elastomer network. Furthermore, widely used acrylate systems are susceptible to oxygen inhibition [15,16,17,18,19]. This inhibition arises because oxygen quenches both the excited states of photoinitiators and the active radical species, forming stable peroxyl radicals that are incapable of re-initiating polymerization, thereby significantly reducing curing efficiency and final conversion [15]. These systems also exhibit significant curing shrinkage [20,21,22,23,24]; for instance, certain acrylate-based monomers can experience volume shrinkage exceeding 20% during photopolymerization, which accumulates during layer-by-layer printing and leads to residual stress, warpage, and dimensional inaccuracies in the final structure [21]. Although thiol–ene systems offer advantages such as low oxygen inhibition and reduced shrinkage [25,26,27], the thiol groups can compete with isocyanate prepolymers in side reactions, disrupting the stoichiometric balance critical for polyurethane network formation [28,29]. In addition, existing systems often rely on expensive raw materials, solvent-borne formulations, and high-temperature curing, limiting their application in large-volume, thick-section materials.

**Figure 1:**
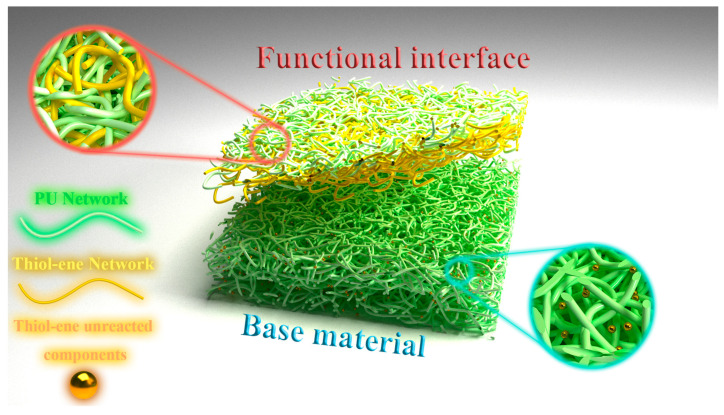
Schematic diagram of the interface mechanism of photocurable durable abrasion-resistant and weather-resistant functionalization.

To address these challenges, this study developed a novel thermal–UV sequential curing hybrid polyurethane system. Poly(tetramethylene ether) glycol (PTMEG) was selected as the soft segment due to its excellent low-temperature flexibility, high elasticity, hydrolysis resistance, and proven utility in durable polyurethane elastomers [30,31,32,33]. As a polyether diol, PTMEG imparts superior hydrolytic stability and chain mobility, which are critical for maintaining mechanical integrity and elasticity in polyurethane systems under physiological or humid conditions [31]. In the hybrid system, two thiols were employed: pentaerythritol tetrakis(3-mercaptopropionate) (PETMP) as a tetra-functional crosslinker to enhance network density and solvent resistance, and 3,6-dioxa-1,8-octanedithiol (DODT) as a flexible chain extender to improve segmental mobility and overall elasticity. Their synergistic use allows independent tuning of crosslinking density and chain flexibility, which is essential for decoupling surface hardness from bulk deformability. A polyurethane prepolymer was synthesized from methylene diphenyl diisocyanate (MDI) and polytetramethylene ether glycol (PTMEG), and 3,3′-dimethoxy-4,4′-diaminobiphenyl (DMTDA) was used as a chain extender to enhance the mechanical properties. By incorporating a thiol–ene photo-click system into the polyurethane network, efficient UV-induced surface functionalization was achieved alongside room-temperature thermal curing. DMTDA exhibited high reactivity with the prepolymer at room temperature, ensuring complete network formation, while the unreacted thiol–ene components acted as chain lubricants, increasing the fracture elongation to 990%. The UV-triggered surface crosslinked layer significantly improved abrasion resistance and environmental durability (Figure 1), maintaining its self-renewing capability even after multiple wear cycles. The obtained homogeneous material combines high flexibility, surface wear resistance, surface optical reconstruction, and dual-shape memory effect, providing a new design strategy for special flexible polyurethane elastomers with wear resistance requirements.

## 2. Materials and Methods

### 2.1. Synthesis

#### 2.1.1. Materials

Diphenylmethane diisocyanate (MDI, 99%). Poly(tetramethylene ether) glycol (PTMEG, also known as hydroxy-telechelic polytetrahydrofuran, Mn = 2000). 4,6-Bis(methylthio)-2-methyl-1,3-benzenediamine (DMTDA). Pentaerythritoltetrakis(3-mercaptopropionate) (PETMP, 98%). 3,6-Dioxa-1,8-octanedithiol (DODT, 95%). 2,4,6-Trisallyloxy-1,3,5-triazine (TAC, 95%). 2,2-Dimethoxy-2-phenylacetophenone (DMPA, 95%). The above materials were purchased from Energy Chemical, Shanghai, China. CUCAT (a catalyst whose primary component is triethylenediamine, 95%) was purchased from Guangzhou Yourun Synthetic Materials Co., Ltd., Guangzhou, China.

#### 2.1.2. Synthesis of Polyurethane Thiol–Ene (PUTE) Polymer Networks

**Scheme 1:**
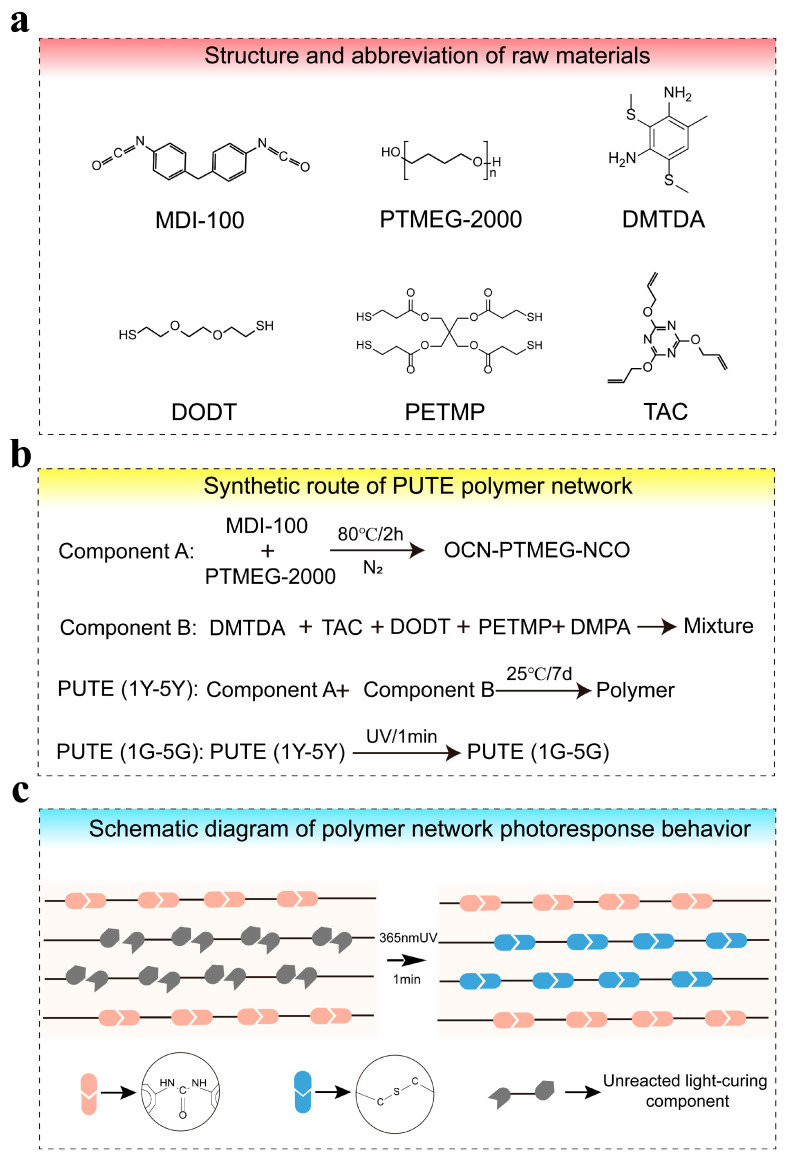
Synthesized path and schematic diagram of PUTE network. (**a**) Structural formula of the raw material for PUTE polymer. (**b**) Synthesis process of PUTE polymer. (**c**) Schematic diagram of the photo-induced self-renewal process of PUTE, where the gray part represents the unreacted photoactive thiol–ene component. The gray dots schematically indicate the photoactive groups (sulfur-based or olefin-based).

The synthetic pathway is illustrated in Scheme 1. A series of PUTE polymers were synthesized by systematically varying the composition of the photocuring system, as detailed in Table 1. The synthesis involves two key steps:

(1) Prepolymer synthesis (Component A): The polyurethane prepolymer (Component A) was synthesized using MDI-100 and PTMEG-2000 as raw materials, with a target NCO content of 6% and a controlled MDI:PTMEG molar ratio of 1:2.32. PTMEG was dried at 110 °C under vacuum for 2 h, then reacted with stoichiometric MDI at 80 °C under N_2_ atmosphere for 2 h to obtain isocyanate-terminated prepolymer (OCN–PTMEG–NCO).
(2) Component B preparation: DMTDA (chain extender), PETMP (tetrafunctional thiol crosslinker), DODT (3,6-dioxa-1,8-octanedithiol, flexible chain extender), TAC (2,4,6-triallyloxy-1,3,5-triazine, multifunctional ene crosslinker), DMPA (2,2-dimethoxy-2-phenylacetophenone, photoinitiator), and CUCAT catalyst were weighed according to Table 1 and thoroughly mixed.

Components A and B were rapidly mixed at room temperature, stirred to homogeneity, and poured into a PTFE mold. The mixture was cured at ambient conditions for 7 days, which was sufficient to achieve stable properties as verified by preliminary experiments, yielding the thermally cured PUTE network (Y-series). For dual-cured samples (G-series), the thermally cured sheets were subsequently exposed to UV irradiation (wavelength: 365 nm, intensity: 250 W/m^2^) for 30 s to induce surface photocrosslinking.

In recent years, the reaction between thiol groups and isocyanates has been widely confirmed as a click reaction [28,29]. The reaction rate of thiol isocyanates is much slower than that of amine isocyanates, so the reaction of amine isocyanates dominates in the initial stage of thermal curing, making thiol groups basically not participate in network formation. To ensure complete reaction of the amine group, the chain extension coefficient of the amine extender is selected as 0.8. After thermal curing, the remaining unreacted thiol and olefin molecules function in the polymer in two main ways: firstly, they act as plasticizers, disrupting crystalline regions and enhancing the fluidity of molecular chains; secondly, on the surface, they participate in subsequent light reactions to form a crosslinked network. Therefore, DODT plays multiple roles throughout the entire process, serving as a partial chain extender, plasticizer, and photoreactive crosslinking agent. This mechanism is consistent with the conclusion drawn in this study.

Therefore, the distinct roles of the photocuring components are as follows: DODT acts as a flexible dithiol chain extender that increases segmental mobility and overall elasticity; TAC serves as a trifunctional ene monomer that undergoes a rapid thiol–ene click reaction with thiol groups upon UV irradiation, forming a densely crosslinked surface layer; DMPA is a radical photoinitiator that generates free radicals under 365 nm light to initiate the thiol–ene reaction. PETMP, a tetrafunctional thiol, contributes to the overall crosslink density and enhances solvent resistance and mechanical integrity. DMTDA functions as a diamine chain extender for the polyurethane network. The synergistic combination of these components enables independent tuning of bulk and surface properties.

### 2.2. Characterization

#### 2.2.1. Fourier Transform Infrared (FTIR) Spectroscopy

Fourier transform infrared (FTIR) spectroscopy was used to monitor the curing reaction of PUTE in order to obtain information about the changes in functional groups. All spectra were recorded in attenuated total reflectance (ATR) mode with 32 scans at a resolution of 4 cm^−1^ in the wavenumber range of 400–3500 cm^−1^ using a Thermo Scientific Nicolet iS5 spectrometer (Waltham, MA, USA).

To qualitatively analyze spectral changes in the polymer samples after aging, FTIR subtraction spectroscopy was employed. The difference spectra were calculated using the following formula:

Difference Spectrum = Aged Spectrum − (Original Spectrum × Subtraction Factor)

This method follows standard subtraction protocols to highlight variations in peak intensity and position before and after treatment. In this study, the subtraction factor was set to 1, representing a direct spectral subtraction. The positive peaks in the resulting difference spectrum indicate an increase in absorbance in the aged sample relative to the original, while negative peaks signify a decrease in absorbance.

#### 2.2.2. X-Ray Photoelectron Spectroscopy (XPS)

X-ray photoelectron spectroscopy (XPS) was performed using a Thermo Scientific K-Alpha spectrometer (Waltham, MA, USA) with a monochromatic Al Kα X-ray source (1486.6 eV). Survey spectra were acquired with 3 scans, while high-resolution S 2p spectra were recorded with 10 scans, at an energy resolution of 0.45 eV. All spectra were analyzed using Avantage software. Background subtraction was carried out using a Smart-type background. The S 2p spectra were deconvoluted into doublets (2p_3_/_2_ and 2p_1_/_2_) with a fixed area ratio of 2:1 and a spin–orbit splitting of 1.18 eV. All spectra were analyzed using Avantage software (Thermo Scientific, Waltham, MA, USA, version 5.992).

#### 2.2.3. Thermogravimetric Analysis and Differential Scanning Calorimetry (TG-DSC)

Thermogravimetric Analysis and Differential Scanning Calorimetry (TG-DSC) was used to analyze the thermal decomposition behavior of PUTE. The testing condition was N2. The temperature range was from room temperature to 800 °C. The rate of temperature increase was 15 °C/min.

#### 2.2.4. Dynamic Mechanical Analysis (DMA)

Dynamic mechanical analysis (DMA) was used to analyze the thermomechanical behavior and damping characteristics of PUTE. L150: DMA test was performed under N_2_. The test mode was dynamic temperature scanning. The temperature range was −50–200 °C. The temperature increase rate was 3 °C/min. The test frequency was 0.4 Hz.

#### 2.2.5. Polymer Swelling Test

The swelling test was used to analyze the crosslinking structure and swelling behavior of PUTE. A sample block was placed in a glass vial and swollen with DMF for 24 h. The original mass of the sample is recorded as m_0_, the swollen mass m_1_, and the extraction of soluble fraction and dried mass m_2_. The gel content (G (%)) and the degree of swelling (S (%)) were calculated according to Equations (1) and (2).

(1) $$G\left(\%\right)=\frac{m_{2}}{m_{0}}$$

(2) $$S\left(\%\right)=\frac{m_{1}}{m_{2}}$$

The network crosslink density ρ is calculated by the Flory–Rehner equation, as shown in Equation (3) [29].

(3) $$\rho =\frac{-[ln\left(1-v_{2}\right)+v_{2}+\chi {v_{2}}^{2}]}{V_{s}\left({v_{2}}^{1/3}-v_{2}/2\right)}\;$$

where V_2_ is the volume fraction of the solubilized polymer and χ is the polymer–solvent interaction parameter, which can be calculated by Equation (4).

(4) $$\chi =0.34+\frac{V_{s}}{RT}{\left(\delta_{\mathrm{polymer}}-\delta_{\mathrm{solvent}}\right)}^{2}$$

where V_s_ is the molar volume of the solvent, R is the gas constant, T is the absolute temperature, δ _polymer_ is the solubility parameter of the polymer, and δ _solvent_ is the solubility parameter of DMF.

#### 2.2.6. Mechanical Properties

An electronic universal tensile testing machine was used to analyze the mechanical properties of PUTE. The tests were carried out at room temperature. The tensile rate was 200 mm/min. Tensile fracture rate and tensile strength index were monitored (JT/T 1039-2016 and GB/T 528) [34,35]. All test specimens were standard dumbbell-shaped specimens (GB/T 1040.3 5B, thickness 2.0 ± 0.1 mm) [36]. The tensile testing machine is model CMT 6000, manufactured by Shenzhen Sansi Testing Equipment Company (Shenzhen, China). All tensile data are reported as mean ± standard deviation. The stress–strain curves in Section 3.2 are representative average curves obtained from three replicate measurements.

#### 2.2.7. Friction and Wear Test

A friction and wear test was used to analyze the wear resistance of PUTE. The test was conducted at room temperature. The test mode was reciprocating mode. Reciprocating amplitude was 5 mm, reciprocating frequency was 20 Hz, ball diameter was 6 mm, applied load was 2 N, abrasion time was 10 min, and mass loss rate and COF coefficient were monitored. The model of the equipment was USA-CETR-UMT 2MT, which is from the Center for Tribology Inc. in Campbell, CA, USA. All experiments were repeated three times.

To investigate the unique self-renewing capability of the UV-cured surface, a sequential abrasion and re-curing process was designed. A bulk polyurethane sample with an initial thickness of approximately 1 cm was used for this study. After each complete wear cycle, a slice approximately 2 mm thick was carefully removed from the top surface of the sample using a precise cutting tool. This newly exposed surface, which still contains unreacted photoactive groups from the initial formulation, was then re-irradiated with 365 nm UV light for 30 s to re-initiate the thiol–ene reaction and form a new wear-resistant layer. This process of abrasion, slicing, and re-curing was repeated for a total of three cycles to evaluate the material’s surface self-renewing capability.

Finally, this study conducted multiple rounds of in situ friction and wear mass loss tests on the 2Y sample, and then conducted ultraviolet irradiation and friction and wear tests again on the 2Y sample that had undergone multiple rounds of friction and wear tests to verify the difference between the subsurface wear resistance of the material and the strategy proposed in this paper. By comparing the inherent wear resistance of the subsurface layer of the material with the wear resistance of the photo-induced wear layer in this article, the usability of the core strategy in this article is further demonstrated, and the interference of the inherent enhancement of material wear resistance caused by the performance difference between the subsurface layer and the surface layer of the material on the strategy in this article is eliminated.

#### 2.2.8. Surface Hardness Test

Shore hardness tests and nanoindentation tests were used to analyze the surface hardness and modulus of PUTE. Nanoindentation tests were performed using a continuous stiffness measurement (CSM) mode with a Berkovich diamond indenter (Agilent G200, Cleveland, OH, USA). The maximum indentation depth was set to 5000 nm, and the applied load was controlled to achieve a strain rate of 0.05 s^−1^. Force–displacement curves were recorded at room temperature. All experiments were repeated three times.

#### 2.2.9. UV Aging Test

UV aging procedure: Samples were aged in a UV aging chamber (wavelength range 290–340 nm) under conditions of 60 °C, 50% relative humidity, and 18 kW rated power. Aging durations: 0 h, 168 h, 360 h, and 720 h. The UV aging oven is model WJ-UVA, manufactured by Dongguan Wanjia Electronic Technology Co., Ltd. (Dongguan, China).

#### 2.2.10. Dynamic Shear Rheology (DSR) Experiments

Dynamic Shear Rheology (DSR) experiments were used to examine the changes in viscoelastic behavior and shear resistance of the materials. A parallel plate (8 mm) rotor and a controlled stress mode (5 N) were used to perform temperature–frequency scans on the polyurethane material. The temperature gradient was set from −10 °C to 65 °C and the frequency gradient was set from 62.8 rad/s to 0.628 rad/s. The specific frequency points are as follows: 62.8, 45.2, 32.5, 23.4, 16.9, 12.1, 8.73, 6.28, 4.52, 3.25, 2.34, 1.69, 1.21, 0.873, 0.628. The instrument model used was Anton Paar Smart Pave, which is from the Anton Paar company in Graz, Austria.

#### 2.2.11. Scanning Electron Microscopy (SEM)

Scanning Electron Microscopy (SEM) was used to analyze the micro-morphology of PUTE. The equipment model was Hitachi Regulus 8100 (Tokyo, Japan), operated at an acceleration voltage of 5 kV. All samples were sputter-coated with a thin gold layer.

#### 2.2.12. Shape Memory Performance Test

Dual-shape memory behavior was evaluated through a four-step thermomechanical cycling procedure. First, the sample was heated to 170 °C (above the high-temperature Tg) and bent to a predefined angle (θ_p1_) under external force, then cooled to 25 °C to fix the temporary shape; the angle after force removal was recorded as θ_f1_. Second, the sample was further cooled to –30 °C (below the low-temperature Tg) and deformed to another angle (θ_p2_) and fixed; the angle was recorded as θ_f2_. Third, the sample was heated to 25 °C to recover the low-temperature programming shape, and the recovered angle (θ_r1_) was measured. Finally, the sample was reheated to 170 °C to recover the original shape, and the final angle (θ_r2_) was recorded. Shape fixation rate (R_f_) and recovery rate (R_r_) were calculated using Equations (5) and (6). The calculation formulas for shape fixation ratio (R*_f_*) and shape recovery ratio (R*_r_*) are as follows:

(5) $$R_{f}(\%)=\frac{\theta_{f}-\theta_{0}}{\theta_{p}-\theta_{0}}\times 100$$

(6) $$R_{r}(\%)=\frac{\theta_{f}-\theta_{r}}{\theta_{f}-\theta_{0}}\times 100$$

Among them, $\theta_{0}$, $\theta_{p}$, $\theta_{f}$, and $\theta_{r}$ represent the initial angle, programming target angle, fixed angle, and restored angle of the sample, respectively. Each sample is tested at least three times, and the results are presented as mean ± standard deviation.

## 3. Results

In the following sections, Group 1 (pure polyurethane, without photoreactive components) and Group 2 (optimal hybrid formulation) are primarily presented for comparative analysis. Group 1 serves as the baseline reference to evaluate the effect of the thiol–ene system, while Group 2 represents the optimized composition exhibiting the best balance of mechanical properties, wear resistance, and self-renewal capability. Group 3 is occasionally included to illustrate the detrimental effects of excessive photoreactive components, such as over-crosslinking and embrittlement.

### 3.1. Analysis and Characterization of PUTE Hybrid Networks

Section 2.1.2 and Scheme 1 illustrate the synthesis pathway and reaction mechanism of the PUTE elastomer. The curing process is divided into two relatively independent stages: thermal curing and photocuring. By uniformly mixing the prepared prepolymer A with component B and curing for seven days at ambient conditions, a PUTE network retaining photoreactive capability is obtained. To characterize the structural features of the polymer network and monitor the reaction progress of thermal and photocuring processes, the following analyses were performed:

#### 3.1.1. FTIR Results Analysis

**Figure 2:**
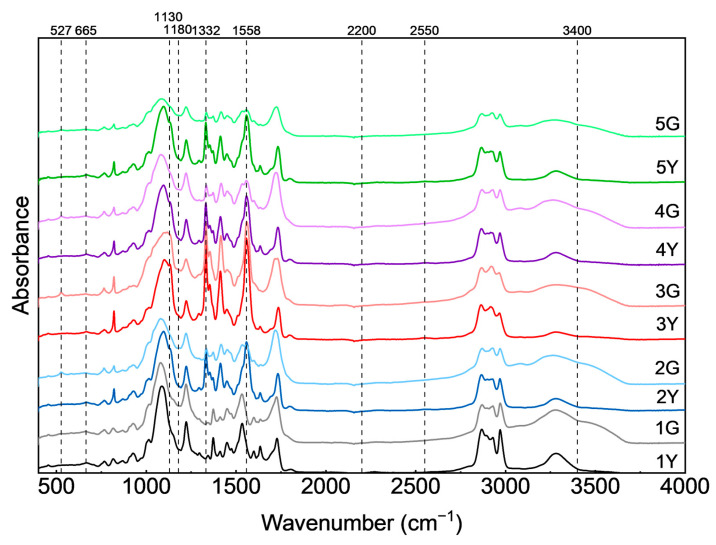
FTIR result graph of PUTE polymer. The dark color curve group represents the Y group that has not undergone photocuring, while the light color curve represents the G group that has undergone photocuring.

FTIR spectroscopy was employed to monitor the curing reactions and identify functional group transformations. Figure 2 shows the FTIR spectra of all Y- and G-series samples. The key characteristic peaks and their assignments are summarized in Table 2, based on the literature [37,38,39,40].

After thermal curing, the complete disappearance of the -NCO stretching vibration at ~2200 cm^−1^ indicates that the isocyanate groups have fully reacted. A broad band at ~3400 cm^−1^ corresponding to -NH and -OH stretching confirms the formation of urethane linkages. The presence of residual thiol groups (-SH, ~2550 cm^−1^) and the triazine ring signals (1332 cm^−1^, 1558 cm^−1^) in the Y-series samples verifies that the photoactive components remain unreacted after thermal curing.

Upon UV irradiation (G-series), significant changes are observed: the intensity of the -SH band decreases, accompanied by the emergence of new peaks at ~665 cm^−1^ (C-S-C stretching) and ~527 cm^−1^ (disulfide -S-S- stretching), evidencing the occurrence of the thiol–ene click reaction and thiol self-coupling. These changes are most pronounced for Group 2 and Group 4 samples, which contain moderate amounts of DODT and TAC. In contrast, Group 3 (high DODT/TAC content) shows a nearly complete consumption of –SH after UV, but also exhibits excessive disulfide formation, indicating competition between the click reaction and oxidative coupling. Group 5 (high DMPA content) displays similar trends, with enhanced photoinitiation efficiency but also increased side reactions, as reflected by stronger disulfide and oxidized sulfur peaks.

The comparison between different formulations reveals that the balance between thiol–ene crosslinking and side reactions is critically influenced by the DODT/TAC ratio and DMPA concentration. The 2Y/2G formulation (DODT/TAC/DMPA = 0.5/0.33/0.5) achieves optimal photocrosslinking efficiency with minimal disulfide byproducts, which correlate well with its superior mechanical and wear-resistant properties discussed later.

#### 3.1.2. Analysis of XPS Results

Spectral analysis (Table S1) reveals the elemental composition (C, O, N, S) of the sample surface. After ultraviolet irradiation, the concentrations of sulfur and oxygen atoms increased, while the concentration of carbon atoms slightly decreased, indicating that oxidation reactions occurred on the surface and carbon chains were cleaved and volatilized.

**Figure 3:**
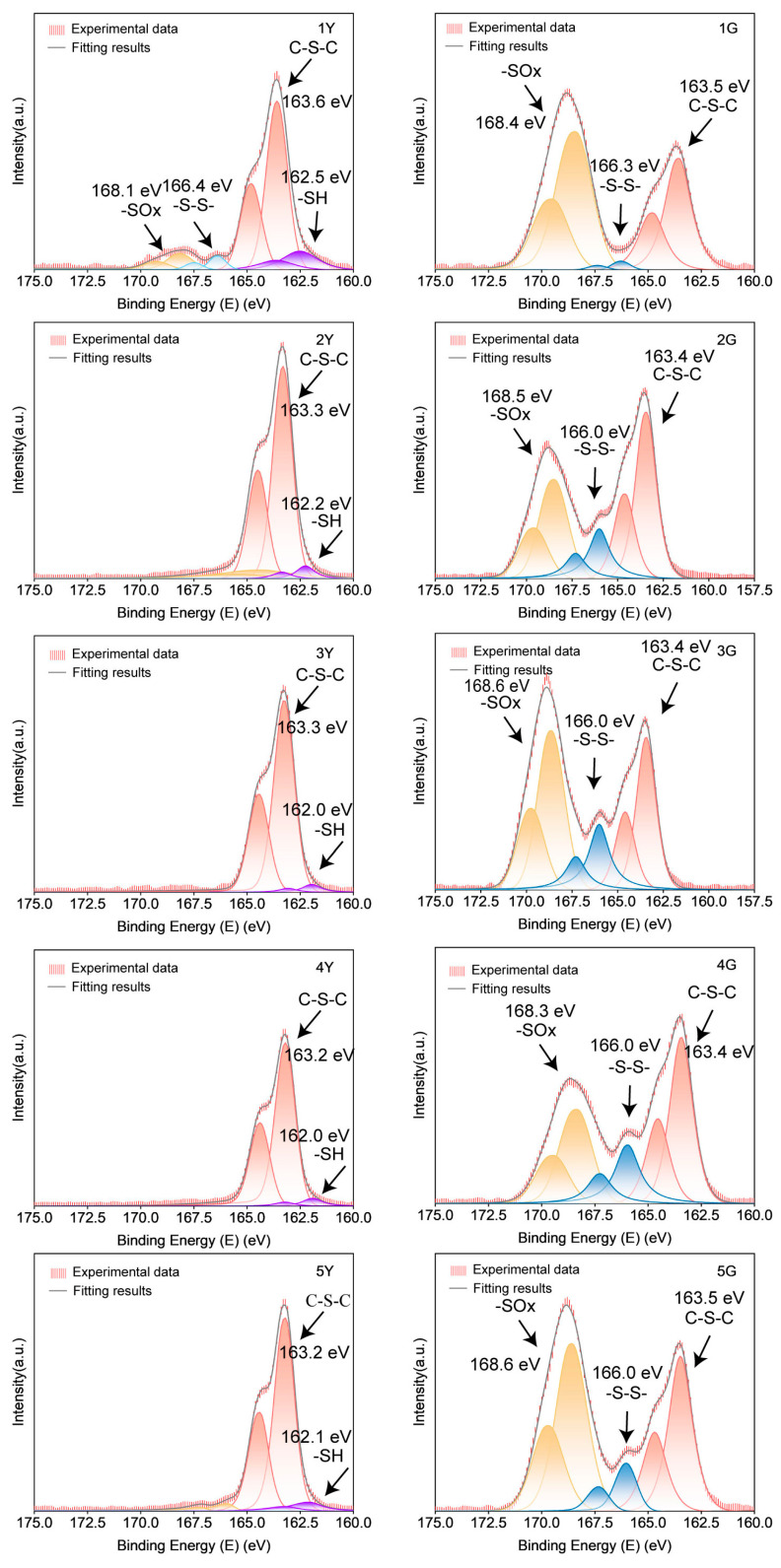
XPS sulfur spectrum of PUTE polymer. The red short vertical lines represent the actual XPS experimental data, while the gray contour lines represent the overall fitting results.

The sulfur in PUTE was analyzed by XPS to determine the sulfur species and reaction pathway before and after UV curing (Figure 3 and Table 3). The results showed that before UV curing, the sulfur in PUTE mainly existed in the form of C-S-C and -SH, which indicated that the UV curing system still had some optical activity after thermal curing [41,42]. During thermal curing, a part of sulfur reacts with the isocyanate group to form a thiocarbamate bond [43]. After UV irradiation, the sulfur-containing functional groups evolve thiol groups (-SH) which are partially consumed to form thioether bonds (C-S-C) via a thiol–ene click reaction, while some –SH groups undergo self-coupling to generate disulfide bonds (-S-S-) [44]. Additionally, a fraction of sulfur is oxidized to sulfonate or sulfate species, as evidenced by the higher binding energy peaks. This shows that mercaptans undergo at least three parallel pathways under light: thiol–ene coupling, thiol self-polymerization and oxidation. Compared with FTIR, thiol–ene components form a photocrosslinking network dominated by thioether and disulfide bonds after illumination, and the oxidation products of sulfur are related to the oxidation of carbon sulfur bonds. Comparative analysis showed that the intensity of the disulfide bond peak (from -SH self-polymerization) was directly related to the content of UV curing components in the system. In contrast, photoinitiator content has little effect on sulfide bond formation. However, excessive photoinitiator promoted more self-polymerization and oxidation of mercaptans, which was proved by the results of samples 4G and 5G. XPS results successfully showed that the formation of photocrosslinked networks was different from polyurethane networks.

From the perspective of material ratio, compared to Group 1 without a photoreactive system, the remaining Groups 2–5 containing photoreactive components exhibited significantly higher C-S bond and -S-S- signal levels after photocuring, indicating that the photocurable components were primarily converted into C-S and -S-S- bonds upon exposure to light, thus proving the formation of a photocrosslinked network. Examining the differences among Groups 2–5, an excessive amount of photocurable components (Group 3) led to the generation of more photooxidation products, thereby reducing the proportion of the C-S bond network. This phenomenon also occurred in the group with an excessive amount of photoinitiator (Group 5). This suggests that an appropriate amount of photocurable components and photoinitiator is crucial for increasing the proportion of the photocrosslinked network.

#### 3.1.3. DMA Results Analysis

**Figure 4:**
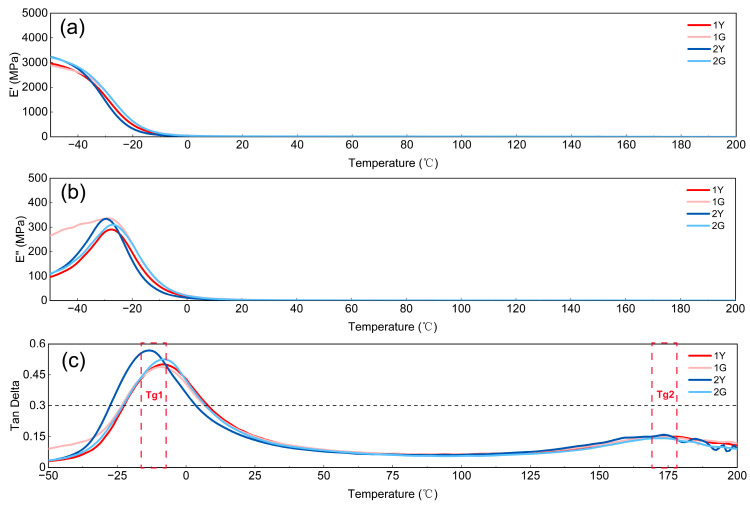
DMA result graph of PUTE polymer (1Y/1G and 2Y/2G), including (**a**) storage modulus (E′), (**b**) loss modulus (E″), and (**c**) loss factor (Tan Delta). TAN Delta greater than 0.3 is generally considered to have good damping performance.

Dynamic mechanical analysis (DMA) was performed to investigate the thermomechanical behavior of PUTE networks. Figure 4 presents the storage modulus (E′), loss modulus (E′′) and loss factor (tan δ) as functions of temperature. Two distinct glass transition temperatures (Tg) are observed: a low-temperature Tg at approximately –10 °C and a high-temperature Tg near 170 °C. The Tg around 170 °C can be partially attributed to the breaking of hydrogen bonds in the polymer and the bond exchange interaction of thiocarbamate bonds [28,45].

DMA results, FTIR and XPS results show that the low-temperature Tg is attributed to the cooperative segmental motion of the polyether soft segments (PTMEG) and the flexible DODT chains. In the Y-series, the presence of unreacted DODT and TAC monomers acts as a molecular lubricant, enhancing chain mobility and slightly lowering the Tg. After UV irradiation (G-series), these monomers are covalently incorporated into the network, restricting segmental motion and causing an upward shift in the low-temperature Tg.

The high-temperature Tg originates from the microphase-separated hard domains composed of urethane linkages. The tan δ peak in this region is broader and less intense for hybrid samples, indicating that the incorporation of thiol–ene components disrupts the ordering of hard segments.

#### 3.1.4. Analysis of TG-DSC Results

**Figure 5:**
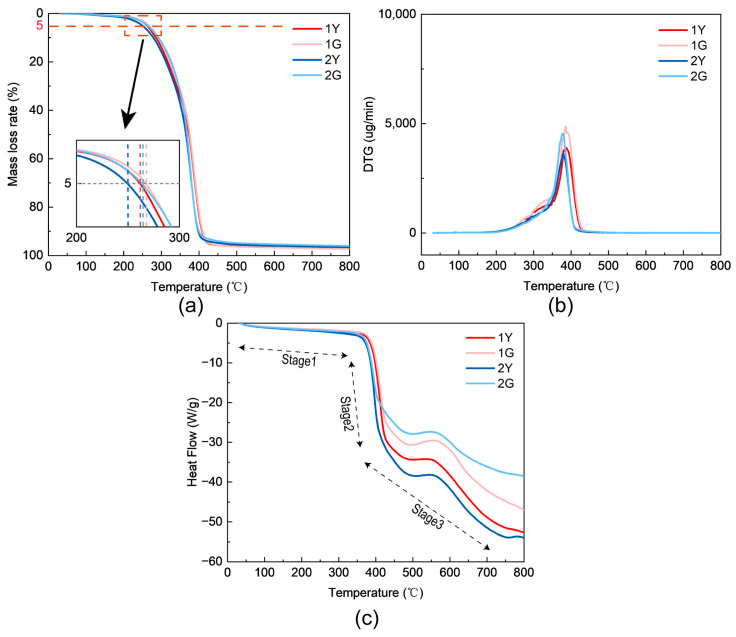
The TG-DSC result graph of PUTE polymer (1Y/1G and 2Y/2G), including (**a**) mass loss rate, (**b**) derivative thermogravimetry (DTG), and (**c**) differential scanning calorimetry (DSC).

Thermogravimetric analysis (TGA) and differential scanning calorimetry (DSC) were employed to investigate the thermal decomposition behavior and stability of the PUTE networks. To clearly demonstrate the impact of the photocrosslinked network on thermal stability, the pure polyurethane (1Y/1G) and the optimal PUTE formulation (2Y/2G) were selected as representative systems. The results are summarized in Figure 5 and reveal distinct degradation stages influenced by the presence of the thiol–ene system and UV curing. Overall thermal stability ranking, as determined by the onset decomposition temperature (Td, onset, defined as the temperature at 5% mass loss), was observed as: 1G > 2G > 1Y > 2Y (Figure 5a,b). This order highlights two key findings: (1) UV exposure generally enhances the high-temperature stability of the cured networks (comparing G-series to their Y-series counterparts), and (2) among the UV-cured samples, the pure PU (1G) exhibits the highest initial stability, while the hybrid system (2G) maintains a significantly elevated stability compared to its uncured state (2Y). The derivative thermogravimetric (DTG) curves (Figure 5b) and DSC thermograms (Figure 5c) allow for a more detailed, multi-stage interpretation of the decomposition process:

Stage 1 (approximately 200–350 °C): This initial mass loss stage is primarily attributed to the thermal scission of sulfide bonds within the thiol–ene network components. For sample 2Y (containing unreacted thiol–ene monomers), an additional mass loss in this region corresponds to the volatilization of free TAC and DODT monomers, which explains its lower overall Td onset compared to 2G. The DSC curve shows a weak endothermic shoulder in this range, consistent with the bond-breaking process of sulfur-based linkages (e.g., -S-S- and -C-S-C-). The presence of these bonds, formed or enriched upon UV curing, acts as a network stabilizer; their sacrificial decomposition at this stage absorbs energy and delays the main-chain degradation [46,47].

Stage 2 (from 350 °C to ~450 °C): This stage represents the primary and most significant mass loss event, corresponding to the decomposition of urethane bonds (-NH-COO-) in the polyurethane backbone. The DTG peak maximum in this region is characteristic of polyurea/polyurethane degradation [48]. The associated strong endothermic peak in the DSC curve confirms this dominant decomposition step. Notably, the TGA traces show that the residual mass at the end of this stage is higher for UV-cured samples (1G, 2G) than for their thermally cured-only counterparts (1Y, 2Y). This indicates that the photocrosslinked thiol–ene network forms a more stable carbonaceous char or interconnected structure that resists complete volatilization, thereby enhancing the overall thermal barrier properties [48].

Stage 3 (above 450 °C): This final stage involves the further oxidative decomposition or carbonization of the remaining char residue. Endothermic events in the DSC may be related to phase changes or further breakdown of aromatic structures from MDI and triazine rings [48].

In conclusion, the TG-DSC analysis provides clear evidence that the UV-induced thiol–ene network significantly alters the thermal degradation pathway of the hybrid material. While introducing a lower-temperature decomposition step associated with sulfur bond cleavage, it concurrently elevates the onset temperature of the main polyurethane decomposition and promotes char formation. This synergistic effect results in a net improvement in the overall thermal stability for the UV-cured hybrid system (e.g., 2G), making it suitable for applications requiring sustained performance at elevated temperatures.

#### 3.1.5. Analysis of Swelling Test Results

**Figure 6:**
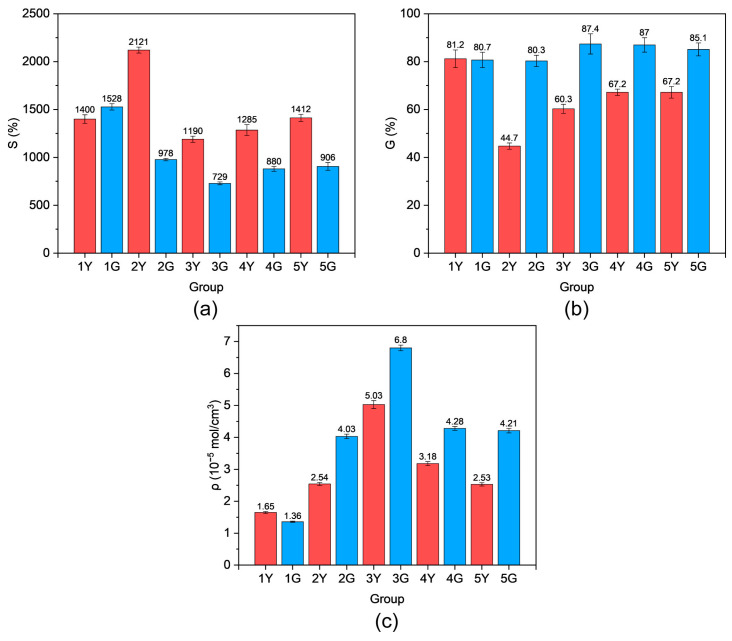
Results of PUTE swelling experiment: (**a**) gel content results, (**b**) the degree of swelling results and (**c**) network crosslink density results.

Swelling tests were conducted to evaluate three critical parameters: gel content (G), swelling ratio (S), and crosslink density (ρ) of the PUTE networks (Table S2 and Figure 6). For samples that did not undergo UV curing (Y-series), higher swelling ratios and lower gel contents are expected, as unreacted monomers and oligomers remain in the network as soluble fractions. This is particularly evident in groups containing photocuring components (Groups 2–5), where the presence of unreacted thiol and ene monomers prior to UV exposure leads to significantly reduced G and elevated S values. After UV irradiation (G-series), these monomers are covalently incorporated into the network via thiol–ene click reactions, resulting in increased gel content, reduced swelling, and enhanced crosslink density. This confirms the formation of a denser, more stable network upon photocuring.

In contrast, for the polyurethane system without photocuring components (Group 1), UV exposure slightly disrupted the existing network, as reflected by a minor decrease in G and ρ and an increase in S. This suggests that in the absence of photoreactive groups, UV irradiation may induce partial chain scission rather than crosslinking.

Comparing groups with different photocuring component contents (Groups 2 and 3), higher concentrations of thiol and ene monomers led to greater improvements in G and ρ after UV exposure. However, even before UV curing, Group 3 exhibited higher crosslink density than Group 2, indicating that excess photoreactive components may undergo spontaneous crosslinking in the absence of light. This unintended pre-crosslinking reduces the controllability of network formation and may compromise material processability.

Analysis of groups with varying photoinitiator (DMPA) contents (Groups 2, 4, and 5) showed that DMPA concentration had minimal impact on the overall crosslink structure, suggesting that the thiol–ene reaction is primarily governed by monomer stoichiometry rather than initiator content under the conditions used. Overall, the swelling results confirm that the thiol–ene system forms a distinct photocrosslinked network dominated by disulfide and thioether bonds, which is structurally different from the polyurethane network and contributes to the enhanced surface properties observed after UV curing.

### 3.2. Mechanical Properties of PUTE Hybrid Networks

**Figure 7:**
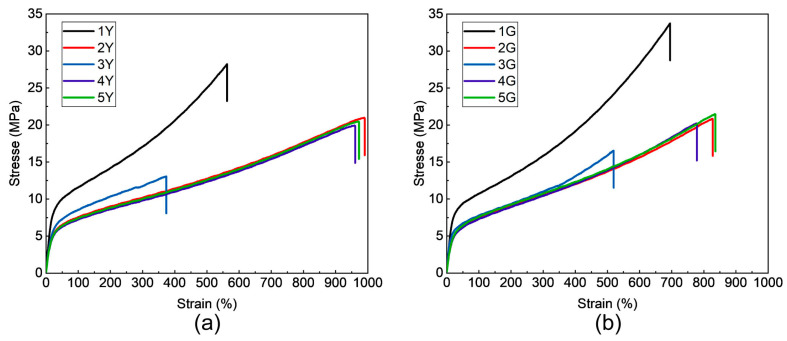
Mechanical properties of PUTE. (**a**,**b**) Direct tensile test results.

**Figure 8:**
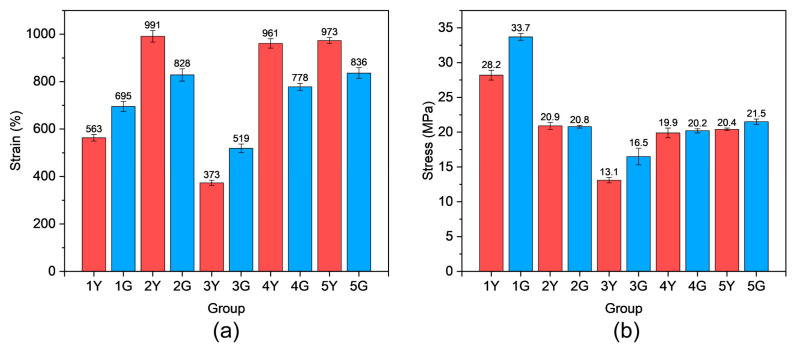
Mechanical properties of PUTE: (**a**) tensile fracture strength of PUTE results; (**b**) elongation at break of PUTE results.

The mechanical properties of PUTE were evaluated via tensile tests (Figure 7 and Figure 8, Table S3). Results demonstrated that incorporating DODT and TAC significantly improved extensibility, achieving a maximum elongation at break of 990%. However, this was accompanied by a reduction in tensile strength (Groups 2, 4, and 5). Excessive DODT, TAC, and DMPA could lead to a drastic decline in mechanical performance, and while post-UV irradiation recovery occurred, the final properties remained unacceptable (Group 3). This is probable owing to unreacted monomers hindering the formation of polyurethane segments, thereby degrading overall performance. After UV exposure, the tensile strength of Groups 2, 4, and 5 remained largely unchanged, but elongation at break slightly decreased. UV irradiation primarily induces thiol–ene click crosslinking of the sample surface, forming a high-modulus layer. This is attributed to the attenuation of light intensity along the depth direction, resulting in a higher degree of crosslinking on the surface compared to the bulk, a well-documented phenomenon in photopolymerization processes [15,21], which evidenced by the increased thioether/disulfide bond content in XPS (Figure 3) and the attenuation of the –SH peak in FTIR (Figure 2). In tensile testing, this hard surface layer preferentially bears the load and restricts the uniform deformation of the underlying soft bulk (polyurethane network), resulting in a slight decrease in macroscopic elongation at break. However, since the tensile strength mainly depends on the load-bearing capacity of the entire sample cross-section, and the thickness of the hard surface layer accounts for a small proportion, its positive contribution to overall strength is offset by the potential negative effect of stress concentration. Therefore, the overall change in tensile strength is not significant. This suggests that UV-induced crosslinking network formation restricted internal segmental deformation and slippage, resulting in reduced extensibility. For the PU system without DODT, TAC, and DMPA (Group 1), UV exposure increased both tensile strength and elongation at break. This may be attributed to short-term UV irradiation partially substituting for conventional post-curing treatments, promoting intermolecular reactions within the material. Since the UV exposure duration was brief, degradation effects caused by prolonged irradiation were negligible.

### 3.3. UV-Induced Abrasion-Resistance of PUTE Hybrid Networks

**Figure 9:**
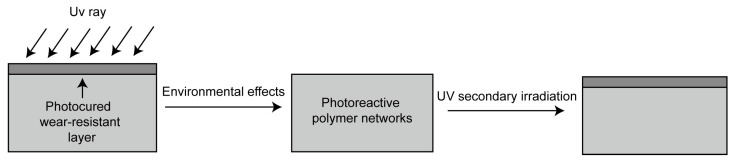
Schematic of PUTE UV-induced wear resistance behavior.

The thiol–ene click reaction facilitates the formation of highly wear-resistant interfaces in PUTE networks. The mechanical robustness of these photo-induced surfaces was evaluated using Shore hardness testing and nanoindentation, providing insight into the network’s photoreactivity and abrasion resistance. Abrasion wear tests were further conducted to directly quantify resistance to mechanical wear. Additionally, DMA, FTIR, and SEM were employed to investigate the weatherability of the interfaces, particularly their resistance to microcracking after prolonged UV aging. Figure 9 shows a schematic diagram of self-renewal behavior after material surface loss under external action.

#### 3.3.1. Shore Hardness and Nanoindentation Test Results

**Figure 10:**
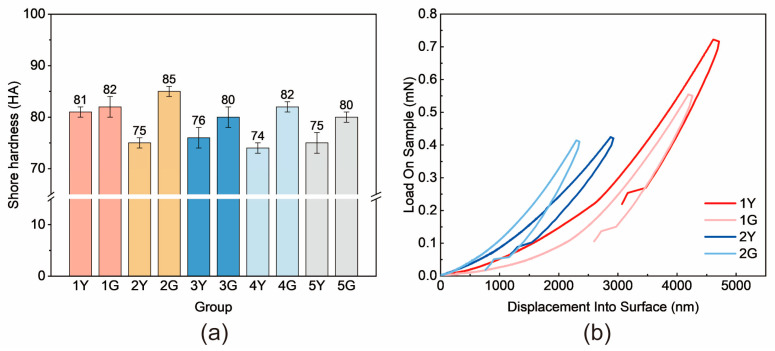
Surface mechanical testing results of PUTE polymer. (**a**) Shore hardness test results; (**b**) nanoindentation test results.

Material hardness serves as a critical indicator for evaluating wear resistance. By analyzing changes in hardness before and after photocuring, the influence of the photocuring system on wear resistance can be directly observed. Shore hardness measurements were conducted to characterize the hardness of PUTE networks (Figure 10a). For the pure PU system (Group 1), UV exposure had minimal impact on hardness, whereas significant differences were observed in other groups, with a maximum hardness change of 10 HA between pre- and post-UV states in Group 2. This indicates that the thiol–ene click-crosslinked network, as expected, enhances the surface hardness and wear resistance of PUTE. While PUTE exhibited reduced surface hardness prior to photocuring, this is acceptable for practical applications since the wear-resistant surface only requires curing on the irradiated side.

Nanoindentation tests provided microscale insights into surface mechanical properties (Figure 10b). Comparing Groups 1 and 2, Group 2 demonstrated consistently higher surface hardness, requiring greater applied force to achieve the same penetration depth. This further confirms the beneficial effect of the thiol–ene photocuring system on surface hardness. Notably, Sample 2Y showed discrepancies between Shore hardness and nanoindentation results: its surface hardness measured via nanoindentation exceeded that of Group 1, conflicting with the Shore hardness data. This discrepancy probably arises because nanoindentation evaluates surface properties at the micrometer scale, where ambient indoor light exposure may have partially cured the surface. However, such curing did not trigger significant photoreactivity in the bulk material.

#### 3.3.2. Friction and Wear Test Results

**Figure 11:**
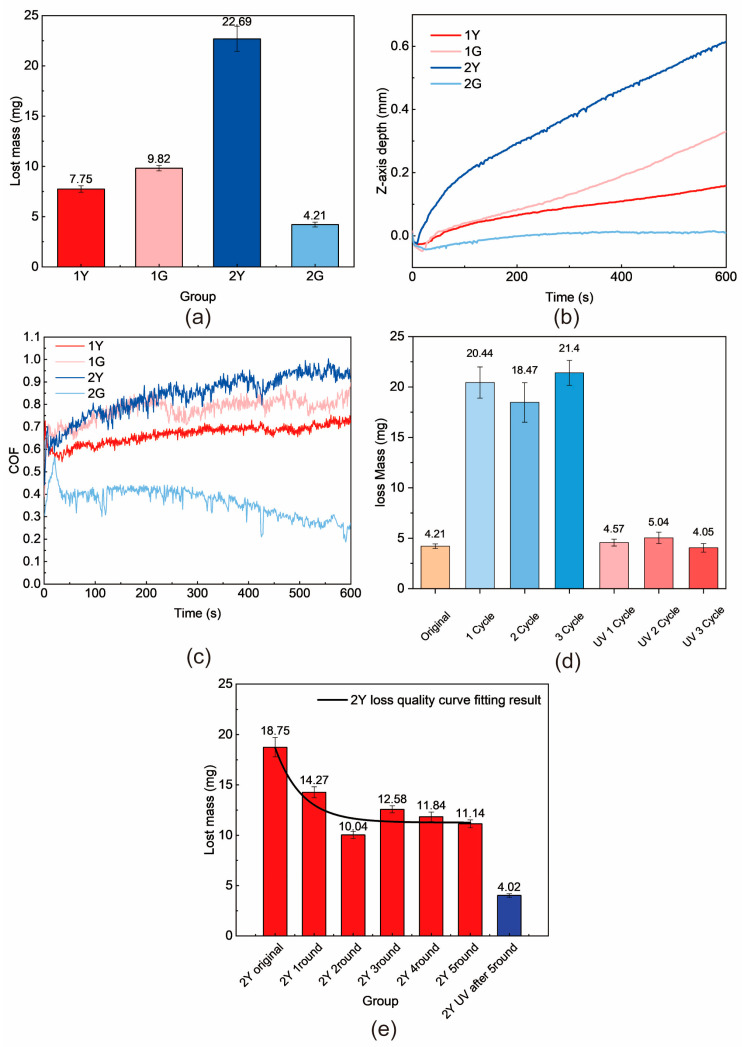
PUTE friction and wear test results: (**a**) lost mass; (**b**) Z-direction wear depth; (**c**) friction coefficient; (**d**) 2G sample cyclic wear test results, where “Original” represents the original value of 2G. Subsequent values followed by cycles indicate the wear test conducted on the newly exposed surface after each peeling. UV followed by cycle numbers signifies that after each wear test on the new surface, it is illuminated again and then subjected to another surface wear test. Each bar graph represents independent data from each wear test. (**e**) Results of multi-round wear and tear mass loss in 2Y group.

Abrasion wear testing was conducted to simulate the abrasive effects of vehicular loading on PUTE networks and directly evaluate the performance of photo-induced wear-resistant surfaces. Key parameters monitored included abrasion mass loss, Z-direction wear depth, and friction coefficient (COF) (Figure 11). Mass loss results (Figure 11a) demonstrated that the photocuring system in PUTE significantly improved surface wear resistance after UV exposure. Sample 2G exhibited a 57% reduction in abrasion mass loss compared to Sample 1G, and an 81% reduction compared to Sample 2Y. Z-direction wear depth (Figure 11b) further corroborated these findings, showing a positive correlation between wear depth and mass loss. Notably, Sample 1G exhibited an increasing wear depth gradient after 10 min of abrasion, whereas Sample 2G maintained nearly constant wear depth, suggesting that most of the mass loss in Sample 2G originated from an initial polishing effect. This hypothesis was supported by COF measurements (Figure 11c): Sample 2G exhibited a steady decrease in COF during abrasion, while COF values for other groups continuously increased. This indicates that Sample 2G lost minimal large-particle debris or bulk polymer fragments during abrasion, whereas other groups shed larger wear particles, increasing surface roughness. This suggests that the wear mechanism on the sample surface has changed after the formation of the photocrosslinked network.

The cyclic wear test on the 2G sample (Figure 11d) further demonstrates the self-renewal capability of this functional layer. After a single test cycle, the top 2 mm surface layer was removed. The newly exposed surface was retested, which was recorded as one cycle. After three cycles, the mass loss rate of the UV-treated experimental group was significantly lower than that of the untreated group. This confirms the repeatability of this photoregenerative functional layer.

The results clearly demonstrate that the thiol–ene click-crosslinked network enhances PUTE’s wear resistance by forming a dense crosslinked polymer network, which effectively resists mechanical abrasion. In contrast, PU materials without the thiol–ene photocuring system (e.g., Group 1) degraded in performance and structural integrity under UV exposure and external loading.

Furthermore, to eliminate the interference of intrinsic changes in the wear resistance properties of the subsurface layer of the material on the strategy proposed in this paper, multiple rounds of in situ wear mass loss tests were conducted on the 2Y group of materials. As shown in Figure 11e, the results indicate that the original 2Y sample shows a fluctuating and decreasing trend in wear quality with an increase in the number of wear cycles after in situ multi-wheel wear. The final wear mass tends to be constant, and the final constant wear mass is approximately 60% of the initial wear mass. This indeed proves that the intrinsic wear resistance of the subsurface layer of the material is superior, which may be due to the more uniform and stable material properties of the subsurface layer. However, compared to the wear quality performance after illumination, the intrinsic wear rate of the subsurface layer is still relatively high, which fully proves the effectiveness of the photo-induced wear resistance strategy in this article. At the same time, further testing was conducted on the worn subsurface under light exposure, and the results showed a further decrease in wear quality.

#### 3.3.3. SEM Test Results

**Figure 12:**
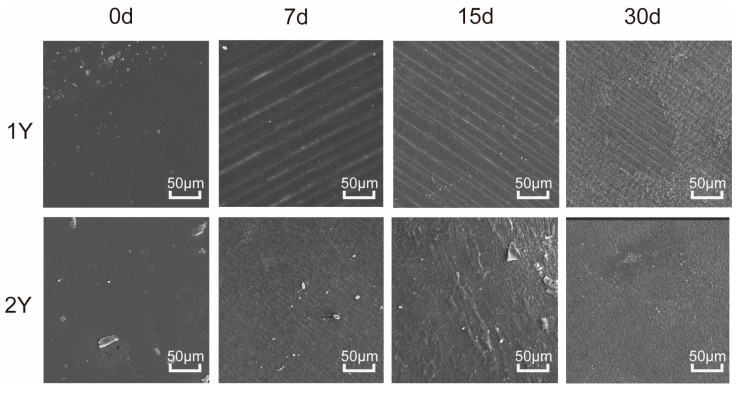
SEM result image of the high strain area of PUTE sample after UV aging.

SEM was employed to examine the surface morphology of PUTE networks after UV aging (Figure 12). The observation regions were selected in high-strain areas near the fracture surfaces of samples after tensile failure, aiming to evaluate the impact of UV aging on the material’s resistance to microcracking under high-strain conditions. Key observations revealed that Sample 1Y exhibited visible microcracks after only 7 days of UV exposure, whereas Sample 2Y maintained surface integrity even after 30 days of UV irradiation. This demonstrates that the thiol–ene crosslinked network significantly enhances UV resistance, preventing microcrack propagation on material surfaces under high-strain conditions and improving the overall weatherability and durability of the polymer.

#### 3.3.4. DSR Test Results

**Figure 13:**
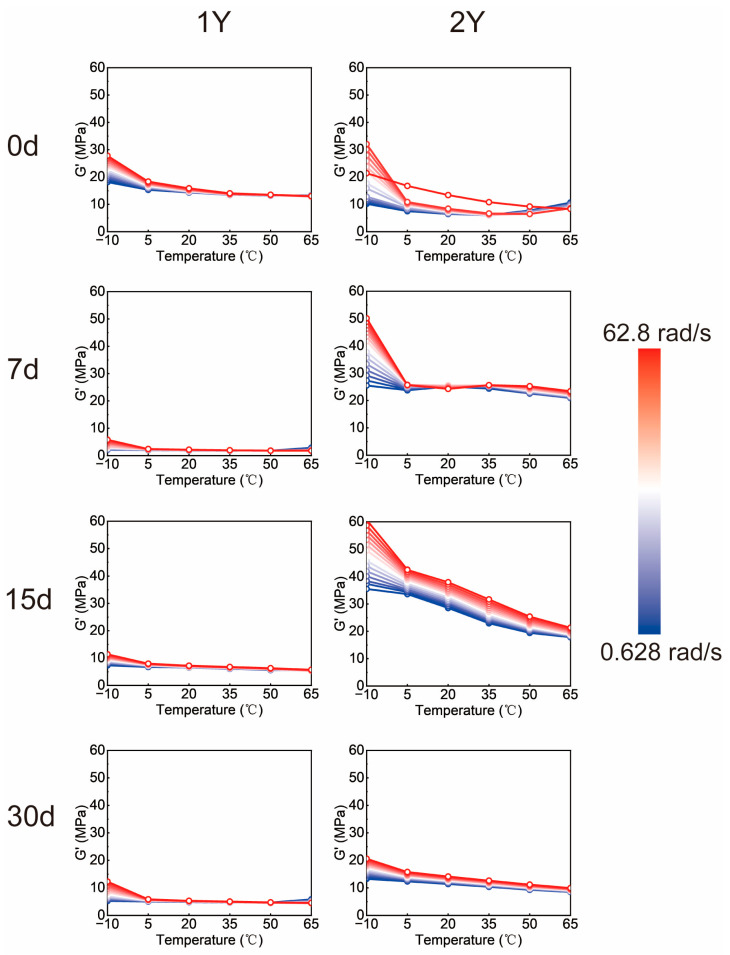
DSR temperature–frequency scanning results of PUTE polymer network after UV aging.

DSR temperature–frequency scans were conducted to evaluate changes in the viscoelastic behavior of PUTE networks during UV aging (Figure 13). Sample 1Y exhibited a significant decline in storage modulus (G′) throughout the UV aging process, indicating a reduction in elastic components of the polymer network. This suggests degradation and structural breakdown of the network, leading to reduced stiffness and integrity. Sample 2Y showed a distinct initial increase followed by a gradual decline in G’ during UV aging. This behavior indicates that the thiol–ene crosslinked network undergoes early-stage crosslinking reactions under UV irradiation, enhancing the overall elasticity and stiffness of the polymer network. This mechanism endows the functional interface with superior weather resistance and damage tolerance. Notably, while Sample 2Y’s G′ decreased over prolonged UV aging (30 days), its final value remained higher than the unaged sample, demonstrating that the thiol–ene photocrosslinked network retains a structurally intact elastic network even after prolonged UV exposure. This underscores its superior UV resistance and durability under long-term environmental stress.

#### 3.3.5. FTIR Test Results

**Figure 14:**
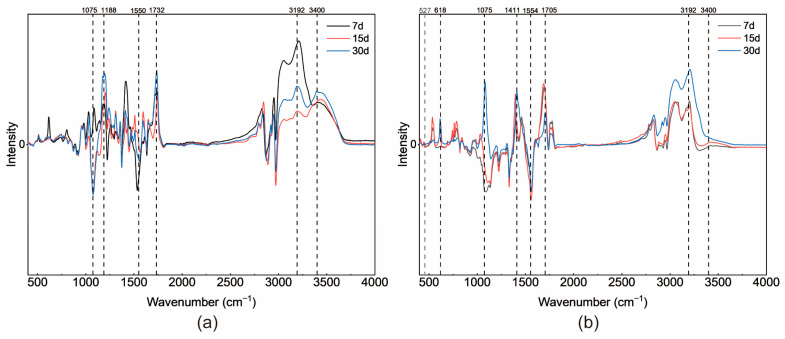
FTIR difference spectrum between the PUTE UV-aged sample for 7d, 15d, 30d and the original sample, with a subtraction factor of 1. (**a**) Group 1 FTIR difference spectrum; (**b**) Group 2 FTIR difference spectrum.

Fourier transform infrared (FTIR) spectroscopy was employed to analyze the chemical evolution of the polyurethane elastomer (PUTE) networks during UV aging (Figure 14). Sample 1Y exhibited evidence of carbon chain oxidation, characterized by an increase in the intensity of characteristic peaks including: 1732 cm^−1^ (C=O stretching vibration), 1188 cm^−1^ (ester C-O-C stretching vibration), and 3400 cm^−1^ (-OH stretching vibration) [38,49,50]. The intensity of these three peaks increased with prolonged UV aging time, indicating that UV aging induces continuous oxidation of the PUTE polymer. Furthermore, the attenuation of the peaks at 1550 cm^−1^ (N-H bending vibration of the urethane groups in polyurethane) and 1075 cm^−1^ (C-O-C stretching vibration) confirmed the partial degradation of the polyurethane (PU) backbone [50,51]. Notably, in contrast to the continuous decrease in the C-O-C bond at 1075 cm^−1^ with increasing UV aging time, the N-H bending vibration at 1550 cm^−1^ exhibited its maximum signal decrease at 7 days of UV aging, followed by a gradual shift in its peak position with further aging. This suggests that during long-term UV aging, the urethane bonds undergo initial degradation, followed by continuous photo-induced side reactions.

Sample 2Y showed minimal evidence of carbon chain oxidation or decomposition. However, new peaks emerged and intensified, including those at 527 cm^−1^ (-C-S- stretching vibration), 618 cm^−1^ (-S-S- stretching vibration), and 1411 cm^−1^ (-SO_3_H stretching vibration), indicating the formation of a sulfur-based crosslinked network. This network compensates for the degradation of the polymer network during UV aging. Unlike the continuous growth of the -S-S- and -SO_3_H peaks with UV exposure time, the -C-S- signal exhibited an initial increase followed by a subsequent decrease. This demonstrates that during prolonged UV aging, -C-S- bonds are initially formed and later undergo further oxidation to other chemical species. Another noteworthy feature is the peak at 1075 cm^−1^ (attributed to C-O-C stretching and S=O stretching). Throughout the UV aging process, this peak’s signal first decreased significantly and then increased rapidly. This suggests severe degradation of the PTMEG carbon chains in the early stages of UV aging, followed by an increase in S=O groups as photooxidation progresses, indicating that the oxidation of sulfur-containing groups becomes the dominant process in the later stages of UV aging [52,53,54].

Both Sample 1Y and Sample 2Y exhibited increased peak intensities at 1732 cm^−1^ and 3192 cm^−1^ (C=O and -OH stretching, respectively) after aging, demonstrating the formation of carboxylic acid groups in both systems [55]. These FTIR results align with the trends observed in the scanning electron microscopy (SEM) and dynamic shear rheology (DSR) analyses, confirming the enhanced UV resistance and structural integrity conferred by the thiol–ene crosslinked network in Sample 2Y.

### 3.4. Heat-Induced Dual-Shape Memory Properties of PUTE Hybrid Networks

**Figure 15:**
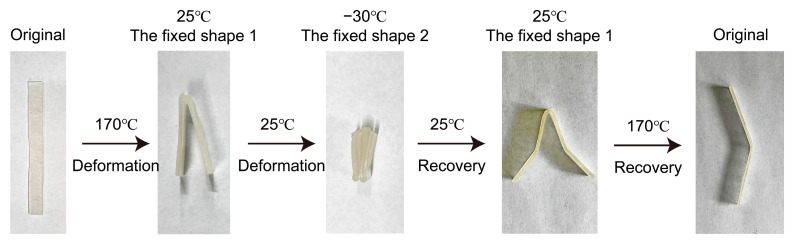
Dual-shape memory process of PUTE network.

**Figure 16:**
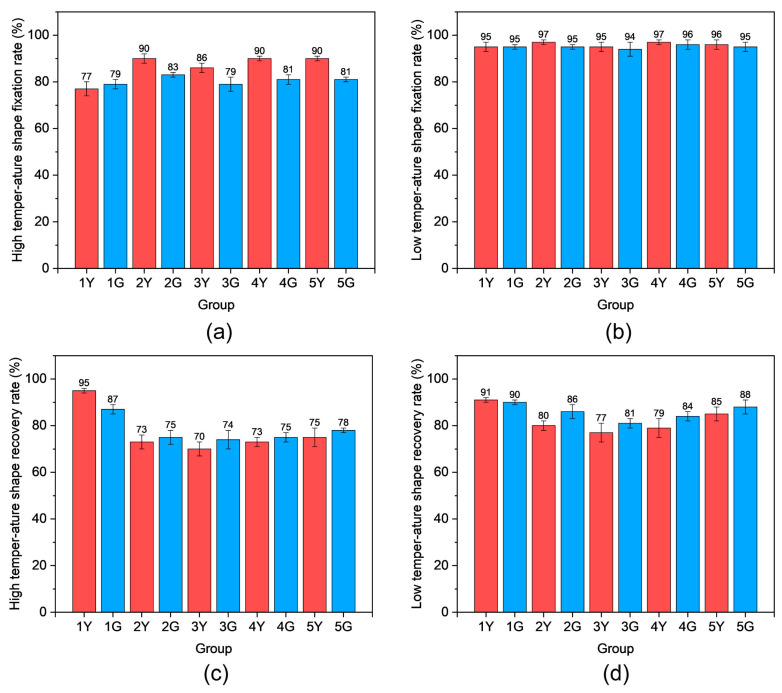
Dual-shape memory process of PUTE results. (**a**) High-temperature shape fixation rate. (**b**) Low-temperature shape fixation rate. (**c**) High-temperature shape recovery rate. (**d**) Low-temperature shape recovery rate.

DMA test results revealed that PUTE exhibits two distinct glass transition temperatures (Tg) at approximately −10 °C and 170 °C (Figure 4), theoretically enabling dual-shape memory behavior. This section investigates the thermally induced dual-shape memory performance of PUTE networks through shape memory experiments (Figure 15 and Figure 16, Table S4). By controlling heating and cooling processes, PUTE can memorize two different shapes and sequentially recover to its original form when the temperature exceeds each Tg threshold, consistent with DMA observations.

Figure 16 summarizes the shape fixing rate and shape recovery rate for Sample 2Y, which serves as the primary matrix material in expansion joint structures. The shape fixing and recovery rates in the low-temperature Tg region (near −10 °C) were significantly higher than those in the high-temperature Tg region (near 170 °C). This superior low-temperature performance is attributed to the crystallization and immobilization of PTMEG and thiol-containing segments below −10 °C. These segments constitute the majority of the PUTE network and exhibit high crystallinity and structural regularity, consistent with DMA results.

In contrast, the shape memory performance at the high-temperature Tg region (170 °C) was notably poorer. This can be attributed to two competing mechanisms. First, at 170 °C, extensive dissociation of hydrogen bonds within the polyurethane network occurs [45], which reduces the physical crosslinking density that typically aids shape fixation and recovery. Second, and more critically, this elevated temperature simultaneously triggers thiourethane bond exchange reactions [28]. Unlike reversible hydrogen bonding, such bond exchange effectively rearranges the covalent network architecture, creating new crosslinks that are unable to relax back to the original configuration upon cooling. This network reconstruction at high temperatures fundamentally compromises the material’s ability to fully recover its permanent shape, explaining why the shape recovery rate in the high-temperature region is the lowest observed.

DMPA content also significantly impacts shape memory performance. With increasing DMPA content, both shape fixing rate and shape recovery rate gradually improve. This enhancement is attributed to more complete curing of the photoreactive network at higher DMPA concentrations during the initial UV exposure, resulting in a more stable and well-defined network structure that minimizes subsequent thermally induced crosslinking reactions at elevated temperatures.

## 4. Discussion

This study demonstrates a successful decoupling of bulk elasticity and surface wear resistance in a homogeneous polyurethane matrix through a sequential thermal–UV curing strategy. The dual-network architecture—comprising a flexible polyurethane network and a photocrosslinked thiol–ene surface layer—is key to achieving this unprecedented combination of properties.

FTIR and XPS analyses confirm that after thermal curing, unreacted thiol and ene groups remain dormant within the matrix. Upon brief UV exposure, these groups undergo a rapid thiol–ene click reaction, generating a dense thioether-crosslinked skin. Concurrently, disulfide bonds form via thiol self-coupling, and minor oxidation produces sulfonate species. The balance between these pathways is governed by the DODT/TAC ratio and DMPA content (Table 1). An optimal composition (2Y/2G: DODT/TAC/DMPA = 0.5/0.33/0.5) yields a high density of thioether linkages with minimal disulfide byproducts, leading to the greatest enhancement in surface hardness and abrasion resistance (81% mass loss reduction) while preserving bulk stretchability (990% elongation). Excessive DODT/TAC (Group 3) causes overconsumption of thiols and excessive disulfide formation, embrittling the surface and compromising bulk integrity. High DMPA content (Group 5) accelerates photoinitiation but also promotes oxidation, slightly reducing wear resistance.

The self-renewing capability originates from unreacted photoactive groups buried beneath the worn surface. After abrasion, fresh thiol and ene moieties are exposed and can be re-crosslinked by a second UV treatment. The friction and wear test shows that the wear resistance of the surface after repeated renewal is similar, confirming the restoration of the protective layer. This cycle can be repeated at least three times without significant loss of performance, a feature rarely reported in conventional polyurethane coatings.

Unlike pure polyurethane (1Y), which undergoes severe chain scission and microcracking upon prolonged UV exposure, the thiol–ene hybrid (2Y) exhibits remarkable photostability. DSR reveals an initial increase in storage modulus due to additional crosslinking, followed by a slow decline that still remains above the unaged value after 30 days. SEM and FTIR confirm the absence of microcracks and suppressed carbonyl/hydroxyl buildup. This resilience is attributed to the sacrificial oxidation of thioether linkages, which dissipates UV energy and delays backbone degradation.

While previous thermo–photo hybrid systems (e.g., acrylate-based) often suffer from oxygen inhibition and high shrinkage, our thiol–ene system offers low shrinkage and minimal oxygen sensitivity [25,26,27]. Moreover, unlike filler-reinforced or coated elastomers, our approach creates a covalently bonded, compositionally graded interface that eliminates delamination risk. The additional dual-shape memory effect further distinguishes this material, enabling programmable deformation for applications such as smart seals or deployable structures.

Despite these advances, challenges remain: (I) the depth of UV penetration limits the self-renewal of deeper defects; (II) the influence of the type and content of materials in the photoreaction system on the properties of materials is not deep enough; (III) long-term stability under combined mechanical and environmental stresses requires further verification. Future work will focus on a more in-depth study of the effects of various light reaction systems on self-renewal function, and will try to introduce dynamic covalent bonds to strengthen and endow more self-renewal function.

## 5. Conclusions

In summary, we have developed a novel polyurethane/thiol–ene hybrid system via a sequential thermal–UV curing strategy that successfully decouples bulk flexibility from surface wear resistance and self-renewal capability. The dual-network design allows independent tailoring of the elastomeric matrix and the photocrosslinked surface layer. The optimal formulation (2Y/2G, with DODT/TAC/DMPA = 0.5/0.33/0.5) achieves an outstanding balance of mechanical properties: tensile strength of 20.9 MPa, elongation at break of 990%, and an 81% reduction in abrasion mass loss after UV curing. The surface layer exhibits self-renewing functionality for at least three wear cycles, enabled by residual photoactive groups that can be re-crosslinked upon re-irradiation. Furthermore, the thiol–ene network imparts exceptional UV aging resistance, suppressing microcrack formation and preserving mechanical integrity even after 720 h of accelerated weathering. The material also demonstrates dual-shape memory behavior with high fixation (>90%) and recovery (>80%) at both low- (−10 °C) and high- (170 °C) transition temperatures. This work provides a paradigm for designing flexible, durable, and smart polymer systems, with promising applications in artificial skin, outdoor flexible components, and adaptive expansion joints.

## Figures and Tables

**Table 1:** Component B raw material ratio.

Group	Subgroup	Mol Equivalence Ratio (vs. n(NCO))				Mass Fraction (%)	
		DMTDA	PETMP	DODT	TAC	DMPA	CUCAT
1	1Y/1G	0.4	0.05	0	0	0	0.5
2	2Y/2G	0.4	0.05	0.5	0.33	2	0.5
3	3Y/3G	0.4	0.05	1	0.66	2	0.5
4	4Y/4G	0.4	0.05	0.5	0.33	0	0.5
5	5Y/5G	0.4	0.05	0.5	0.33	4	0.5

**Table 2:** Key characteristic peak position diagram in FTIR results of PUTE polymer.

Wavenumber (cm^−1^)	Corresponding Functional Group
~527	Disulfide bond (-S-S-)
~665	C-S-C group
~1332	In-plane C-N stretching vibration of the triazine ring
~1558	C=N stretching vibration in the triazine ring
~2200	Isocyanate group (-NCO)
~2550	Thiol group (-SH)
~3400	Amine group (-NH_2_, from DMTDA)/possible oxidation by-products (e.g., -OH, -NH)
~2950, ~2870	C-H stretching (CH_2_, CH_3_)
~3050	Aromatic C-H stretching
~1700	-C=O
~1103	PTMEG -C-O-C-

**Table 3:** The areas of various bonds in S 2p3 region of XPS.

	-SH	C-S-C	-S-S-	-SO_x_
1Y	1946	9785	782	1389
1G	-	14,724	717	23,215
2Y	1819	29,123	-	-
2G	-	19,657	8011	15,210
3Y	1008	19,263	-	-
3G	-	16,782	10,355	22,857
4Y	1617	24,131	-	-
4G	-	18,705	9033	14,184
5Y	3201	31,572	-	-
5G	-	18,162	5145	24,472

## Data Availability

The original contributions presented in this study are included in the article/Supplementary Materials. Further inquiries can be directed to the corresponding authors.

## Supplementary Materials

The following supporting information can be downloaded at https://www.mdpi.com/article/10.3390/ma19071366/s1. Table S1. PUTE element content (C, O, N, S, Si). Table S2. Swelling test parameters. Table S3. Mechanical performance parameter results. Table S4. Dual shape memory recovery rate and fixation rate of PUTE network.
