# Supplementary material for: UV-Induced Self-Renewing Wear-Resistant Flexible Polymer from a Polyurethane/Thiol–Ene Hybrid System
Source: Materials (Basel). 2026 Mar 30;19(7):1366. doi: 10.3390/ma19071366 (PMC13075023; doi:10.3390/ma19071366)
Supplement: Supplementary file 1 [file materials-19-01366-s001.zip › materials-4181290-supplementary.pdf]

Table S1. PUTE element content (C, O, N, S, Si).

	C	O	N	S	Si
1Y	34.77%	34.77%	2.57%	2.03%	2.41%
1G	25.41%	41.58%	3.48%	4.46%	2.32%
2Y	32.82%	33.63%	3.99%	4.54%	2.33%
2G	26.80%	38.93%	4.18%	5.40%	2.00%
3Y	39.80%	28.06%	3.26%	3.41%	2.54%
3G	27.00%	38.83%	3.70%	5.28%	2.33%
4Y	39.13%	29.42%	2.99%	3.47%	2.09%
4G	27.21%	38.71%	3.45%	5.14%	2.55%
5Y	32.78%	33.60%	4.17%	4.67%	2.12%
5G	25.19%	40.02%	4.11%	5.85%	2.13%

Table S2. Swelling test parameters.

Group	G (%)	S (%)	ρ (10^{-5} mol/cm^3)
1Y	81.2±3.7	1400±46	1.65±0.03
1G	80.7±3.2	1528±33	1.36±0.02
2Y	44.7±1.3	2121±31	2.54±0.05
2G	80.3±2.4	978±12	4.03±0.07
3Y	60.3±1.9	1190±32	5.03±0.12
3G	87.4±4.2	729±15	6.80±0.09
4Y	67.2±1.3	1285±57	3.18±0.06
4G	87.0±3.0	880±25	4.28±0.06
5Y	67.2±2.4	1412±38	2.53±0.05
5G	85.1±2.7	906±40	4.21±0.07

Table S3. Mechanical performance parameter results.

**Group**	**Tensile property**	
	**Stress (MPa)**	**Strain (%)**
1Y	28.2±0.7	563±14
1G	33.7±0.5	695±21
2Y	20.9±0.5	991±23
2G	20.8±0.2	828±26
3Y	13.1±0.4	373±11
3G	16.5±1.2	519±18
4Y	19.9±0.7	961±20
4G	20.2±0.3	778±15
5Y	20.4±0.2	973±13
5G	21.5±0.4	836±23

Table S4. Dual shape memory recovery rate and fixation rate of PUTE network.

**Group**	**High temperature shape fixation rate (%)**	**Low temperature shape fixation rate (%)**	**High temperature shape recovery rate (%)**	**Low temperature shape recovery rate (%)**
1Y	77±3	95±2	95±1	91±1
1G	79±2	95±1	87±2	90±1
2Y	90±2	97±1	73±3	80±2
2G	83±1	95±1	75±3	86±3
3Y	86±2	95±2	70±3	77±4

3G	79±3	94±3	74±4	81±2
4Y	90±1	97±1	73±2	79±4
4G	81±2	96±2	75±2	84±2
5Y	90±1	96±2	75±4	85±3
5G	81±1	95±2	78±1	88±3
